# The association between serum sodium level and tuberculous meningitis compared with viral and bacterial meningitis

**DOI:** 10.1038/s41598-021-90358-5

**Published:** 2021-05-25

**Authors:** Seunghee Na, Taewon Kim, In-Uk Song, Sung-Woo Chung, Seong-Hoon Kim, Yoon-Sang Oh, Juhee Oh, Woojun Kim

**Affiliations:** 1 Department of Neurology, Incheon St. Mary’s Hospital, The Catholic University of Korea, Seoul, 06591 Korea; 2 Department of Neurology, Uijeongbu St. Mary’s Hospital, The Catholic University of Korea, Seoul, Korea; 3 Department of Neurology, Yeouido St. Mary’s Hospital, The Catholic University of Korea, Seoul, Korea; 4 Department of Neurology, St. Vincent’s Hospital, The Catholic University of Korea, Seoul, Korea; 5 Department of Neurology, Seoul St. Mary’s Hospital, The Catholic University of Korea, Seoul, Korea

**Keywords:** Microbiology, Diseases, Neurology

## Abstract

We evaluated the association between hyponatremia and tuberculous meningitis (TBM) with the aim of providing additional information for differential diagnosis from other types of infectious meningitis, especially viral meningitis (VM). Cross-sectional and longitudinal data involving 5026 participants older than 18 years were analyzed in the total population and a propensity-matched population. The initial and lowest sodium levels and longitudinal changes in TBM, bacterial meningitis (BM), and VM patients were compared. Participants in the TBM group were enrolled when they were diagnosed as possible, probable, or definite TBM according to the Marais’ criteria. The initial serum sodium level was significantly lower in TBM patients than in BM and VM patients (136.9 ± 5.9 vs. 138.3 ± 4.7 mmol/L, *p* < 0.001 for TBM vs. BM, and 139.0 ± 3.1, *p* < 0.001 for TBM vs. VM), and it decreased significantly more steeply to lower levels in both the TBM and BM patients compared with VM patients. The lowest serum sodium level was in the order of TBM < BM < VM patients, and the change was statistically significant in all subgroups (131.8 ± 6.4, 133.1 ± 5.1, 137.4 ± 3.7, respectively, *p* < 0.001). Participants with lower serum sodium level were more likely to have a diagnosis of TBM rather than VM, and this association was more pronounced for the lowest sodium level than the initial sodium level [OR 4.6 (95% CI 2.4–8.8, *p* < 0.001)]. These findings indicate that baseline and longitudinal evaluation of serum sodium level can provide information for differential diagnosis of TBM from BM or VM.

## Introduction

Tuberculosis remains a global health problem, with an estimated 10 million cases and 1.4 million deaths worldwide in 2019^1^. Tuberculous meningitis (TBM) accounted for 6% of the 57,217 extrapulmonary tuberculosis cases in a large epidemiology study^2^. Even considering that TBM is difficult to diagnosis, and the incidence rate might be underestimated, the global burden of TBM is estimated as at least 100,000 cases per year^3^. TBM is the most lethal form of tuberculosis, resulting in death or severe disability in approximately 50% of the affected patients^4^. In contrast, most aseptic viral meningitis (VM) patients have a self-limiting, good prognosis without any neurological sequelae. Therefore, rapid differential diagnosis of TBM from other types of infectious meningitis and prompt optimal treatment are crucial in preventing the devastating neurological sequelae of TBM, including cranial nerve dysfunction, hydrocephalus, and vascular complications including stroke.

Despite the critical need for distinguishing TBM from other types of infectious meningitis, the diagnosis of TBM often is challenging and delayed by inconclusive laboratory results because of the low sensitivity and slow speed of conventional bacteriology tests. Microscopy and Ziehl–Neelsen staining to detect acid-fast bacilli has a 50% sensitivity for high bacterial burden tuberculosis, such as cavitary pulmonary tuberculosis, and shows 10–20% sensitivity for paucibacillary diseases such as TBM^3^. Although cultures for *Mycobacterium tuberculosis* are more sensitive than microscopy, they usually take 2–3 weeks and require a biosafety level 3 laboratory. The culture sensitivity for TBM is low at 5–58% depending upon the kind of media used and the testing facility^5,6^, and in the sensitivity of the conventional Lowenstein-Jensen culture is low at 10.9%^7^. Diagnosis of TBM is difficult because of similar cerebrospinal fluid (CSF) profiles between VM and TBM, as well as the indolent clinical onset with initial non-specific symptoms^8^ that are indistinguishable from the early symptoms of VM. The CSF profile of TBM typically shows lymphocytic pleocytosis, which is similar to the profile of aseptic meningitis including VM. Although CSF analysis of TBM typically shows lower glucose and higher protein concentrations compared with findings in VM, these changes could not be evident at the early stage of disease.

Hyponatremia is a common finding in acute brain disease^9^. It is common in patients with traumatic brain injury, subarachnoid hemorrhage, and brain tumors as well as patients who have undergone intracranial procedures^10^. Hyponatremia has been reported to be associated with septic meningitis including bacterial meningitis (BM) and TBM^11–13^.

In this study, we evaluated the association between hyponatremia and TBM with the aim of providing information for differential diagnosis of TBM from other types of infectious meningitis, especially VM.

## Results

### Baseline analysis

The baseline characteristics of the patients analyzed in this study are summarized in Table 1. In total, data from 5026 participants were analyzed. For blood test results, data from 4963 participants were available for analysis of WBC count, 4925 participants for analysis of LDH level, 1028 participants for analysis of procalcitonin level, and 4962 participants for analysis of sodium level. In CSF analysis, 2797 participants were included in the analysis of WBC count, 3147 participants in analysis of glucose, 3076 participants in analysis of LDH level, 3193 participants in analysis of protein levels, 1339 participants in analysis of tuberculosis cultures, 1852 participants in analysis of tuberculosis PCR results, and 434 participants in analysis of VZV PCR results.

**Table 1:** Baseline clinical characteristics and laboratory findings in patients with tuberculosis, viral, and bacterial meningitis.

	TBM	BM	VM	*p* value			
	n = 295	n = 130	n = 4601	Among three groups	TBM versus BM	BM versus VM	VM versus TBM
Age (year), mean ± SD	44.7 ± 18.6	52.1 ± 18.8	35.6 ± 14.2	< 0.001**	< 0.001**	< 0.001**	< 0.001**
Female, n (%)	118 (40.0)	56 (43.1)	2295 (49.9)	< 0.001**	0.552	0.126	0.001**
Blood							
Serum WBC, × 10^3^/μL (n)	9.0 ± 6.1	13.2 ± 11.5	8.7 ± 4.1 (4538)	< 0.001**	< 0.001**	< 0.001**	0.728
LDH, U/L (n)	503.8 ± 1078.0 (295)	460.1 ± 268.2 (125)	387.2 ± 627.9 (4505)	0.007**	0.823	0.473	0.013*
Procalcitonin, ng/ml (n)	3.5 ± 16.2 (122)	7.1 ± 19.0 (63)	1.5 ± 9.0 (843)	< 0.001**	0.404	0.056	0.360
HIV Ag/Ab positive, n (%)	1 (0.3%)	0	4 (0.1%)	0.386			
Initial Na level, mmol/L (n)	136.9 ± 5.9 (295)	138.3 ± 4.7 (130)	139.0 ± 3.1 (4537)	< 0.001**	0.041*	0.264	< 0.001**
Lowest Na level, mmol/L (n)	131.8 ± 6.4 (295)	133.1 ± 5.1 (130)	137.4 ± 3.7 (4537)	< 0.001**	0.061	< 0.001**	< 0.001**
**CSF**							
WBC (/μL)	882.0 ± 7161.1 (285)	1276.5 ± 2150.7 (112)	368.5 ± 7797.4 (2797)	0.279	0.786	< 0.001**	0.584
Neutrophil (%)	18.9 ± 27.4	65.5 ± 31.2	15.4 ± 25.6	< 0.001**	< 0.001**	< 0.001**	0.198
Lymphocyte (%)	70.8 ± 31.7	21.8 ± 26.5	52.3 ± 40.1	< 0.001**	< 0.001**	< 0.001**	< 0.001**
Eosinophil (%)	1.0 ± 3.2	0.7 ± 1.2	1.8 ± 7.3	0.427	0.817	0.027	0.305
Monocyte (%)	7.7 ± 11.5	7.9 ± 7.9	7.8 ± 9.6	0.992	0.999	0.999	1.000
Glucose, mg/dL (n)	51.9 ± 23.5 (280)	55.8 ± 40.9 (105)	62.2 ± 24.8 (2762)	< 0.001**	0.363	0.114	< 0.001**
LDH, U/L (n)	229.8 ± 1262.9 (272)	225.0 ± 389.9 (98)	61.5 ± 249.0 (2706)	< 0.001**	1.000	< 0.001**	0.085
Protein, mg/dl (n)	306.6 ± 795.7 (283)	240.1 ± 266.9 (112)	78.8 ± 244.0 (2798)	< 0.001**	0.516	< 0.001**	< 0.001**
CSF tuberculosis culture, positive rate, n (%)	35/279 (12.5)	0/61 (0)	1/1112 (0.1)				
CSF tuberculosis PCR, positive rate, n (%)	30/262 (11.4)	0/75 (0)	1/1515 (0.1)				
Xpert® MTB/RIF, positive rate, n (%)	29/215 (13.5)	0/60 (0)	0/1322 (0)				
CSF VZV PCR, positive rate, n (%)	2/56 (3.6)	0/19 (0)	45/359 (12.5)				
Mortality at 90 days, n (%)	27 (9.2)	19 (14.6)	61 (1.3)	< 0.001**			

BM, bacterial meningitis; CSF, cerebrospinal fluid; LDH, lactate dehydrogenase; PCR, polymerase chain reaction; TBM, meningitis; VM, viral meningitis; VZV, varicella-zoster virus; WBC, white blood cells.

All scores are shown as percent, n value, or mean (SD).

(n): number of patients who underwent the test.

Analyses were performed by analysis of variance (ANOVA), independent sample t-test, Fisher’s exact test, or χ2 test.

*p < 0.05, **p < 0.01.

Age was significantly different between the three subgroups (TBM vs. BM vs. VM, 44.7 ± 18.6 vs. 52.1 ± 18.8 vs. 35.6 ± 14.2 years, *p* < 0.001). There were more females in the VM group than in the TBM group (49.9% vs. 40%, *p* = 0.001). Serum WBC count was significantly higher in the BM group than in the TBM and VM groups (13.2 ± 11.5 vs. 9.0 ± 6.1 vs. 8.7 ± 4.1 × 10^3^/μL, *p* < 0.001). Serum LDH level was significantly higher in the TBM group than in the VM group (503.8 ± 1078.0 vs. 387.2 ± 627.9 U/L, *p* = 0.013). Serum procalcitonin level had a strong trend to be higher in the BM group than in the VM group (7.1 ± 19.0 vs. 1.5 ± 9.0 ng/ml, *p* = 0.056). In CSF analysis, WBC count was significantly different between the BM and VM groups (1276.5 ± 2150.7 vs. 368.5 ± 7797.4, *p* < 0.001), and glucose level was significantly lower in the TBM group than in the VM group (51.9 ± 23.5 vs. 62.2 ± 24.8 mg/dL, *p* < 0.001). The VM group had lower LDH level than the BM group (61.5 ± 249.0 vs. 225.0 ± 389.9 U/L, *p* < 0.001), and the protein levels were significantly lower in the VM group than in the TBM and BM groups (78.8 ± 244.0 vs. 306.6 ± 795.7 vs. 240.1 ± 266.9 mg/dl, respectively, *p* < 0.001). In the TBM group, the positive rates of CSF tuberculosis cultures and PCR tests were 12.5% and 11.4%, respectively. The positive rate of Xpert® MTB/RIF was 13.5%. Among the 295 TBM patients, 39 (13.2%) were diagnosed as definite TBM, 112 (38%) were diagnosed as probable TBM, and 144 (48.8%) were diagnosed as possible TBM.

VZV PCR in the CSF was positive in 3.6% of the TBM group compared with 12.5% of the VM group (*p* = 0.049).

### Initial and lowest serum sodium levels: cross-sectional analysis

The initial serum sodium level was significantly lower in the TBM group compared with the BM and VM groups (136.9 ± 5.9 vs. 138.3 ± 4.7 vs. 139.0 ± 3.1 mmol/L, *p* < 0.001 and *p* = 0.041, respectively), but there was no difference between the BM and VM groups. In analysis of the lowest serum sodium level, the significant difference between the TBM and VM groups was maintained (131.8 ± 6.4 vs. 137.4 ± 3.7 mmol/L, *p* < 0.001), while the sodium level was significantly lower in the BM group compared with the VM group (133.1 ± 5.1 vs. 137.4 ± 3.7 mmol/L, *p* < 0.001) (Table 1). However, there was no difference in lowest sodium level between the TBM and BM groups.

The average numbers of days from initial sodium level to lowest serum sodium level were 4.9 ± 6.0, 4.5 ± 7.0, and 0 ± 3.1 days in the TBM, BM, and VM groups, respectively.

We performed propensity-matched population analysis by balancing according to age, sex, and CSF profiles including WBC, glucose, and protein (Table 2). The initial serum sodium level in the three subgroups showed the same significant difference pattern as in analysis of the total population. The initial serum sodium level was significantly lower in the TBM group compared with the BM and VM groups (TBM vs. BM, 136.7 ± 7.0 vs. 138.3 ± 5.0 mmol/L, *p* = 0.040; TBM vs. VM, 136.9 ± 5.9 vs. 138.5 ± 9.1, *p* = 0.013), but no difference was found between the BM and VM groups. The lowest serum sodium level was in the order of TBM < BM < VM. All comparisons among the three subgroups were statistically significant (TBM vs. BM, 130.9 ± 7.5 vs. 132.7 ± 5.3 mmol/L, *p* = 0.037; BM vs. VM, 132.7 ± 5.3 vs. 135.2 ± 5.7 mmol/L, *p* = 0.001; TBM vs. VM, 131.7 ± 6.5 vs. 135.4 ± 9.6 mmol/L, *p* < 0.001).

**Table 2:** Comparison of initial and lowest serum sodium levels in propensity-matched populations with tuberculous, viral, and bacterial meningitis.

	TBM versus BM			BM versus VM			VM versus TBM		
	TBM	BM	*p* value	BM	VM	*p* value	VM	TBM	*p* value
Propensity-matched population	n = 105	n = 105		n = 105	n = 105		n = 276	n = 276	
Initial Na level (mmol/L)	136.7 ± 7.0	138.3 ± 5.0	0.040*	138.3 ± 5.0	139.0 ± 4.2	0.291	138.5 ± 9.1	136.9 ± 95.9	0.013
Lowest Na level (mmol/L)	130.9 ± 7.5	132.7 ± 5.3	0.037*	132.7 ± 5.3	135.2 ± 5.7	0.001**	135.4 ± 9.6	131.7 ± 6.5	< 0.001**

BM, bacterial meningitis; TBM, tuberculous meningitis; VM, viral meningitis.

All scores are shown as percent, n value, or mean (SD).

The propensity score was matched 1:1 between the two comparison groups controlling for age, sex, cerebrospinal fluid (CSF) white blood cell count, CSF glucose, and CSF protein. The analyses were performed with the independent t-test.

*p < 0.05, **p < 0.01.

### Analysis of changes in serum sodium level by repeated-measures ANOVA

**Figure 1:**
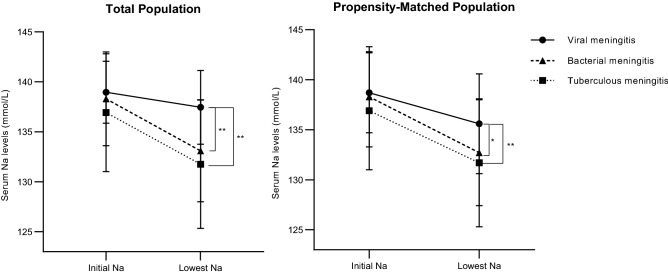
Changes in serum sodium level in VM, BM, and TBM groups. P values for the grou*p* × time interactions are demonstrated. *p < 0.05, **p < 0.01.

We next analyzed grou*p* × time interactions (reflecting whether there was a significant influence of meningitis subtype on interval changes in serum sodium level) (Fig. 1). In both the total and propensity-matched populations, there was significant grou*p* × time interaction between meningitis subtype and interval change in serum sodium level, which significantly decreased more steeply to a lower level in both the TBM and BM groups compared with the VM group. However, no grou*p* × time effect was found between the TBM versus BM groups (in the total population, the p-values of the grou*p* × time effect were *p* = 0.959, *p* < 0.001, and *p* < 0.001 for TBM versus BM, BM versus VM, and TBM versus VM, while in the propensity-matched population analysis, they were *p* = 0.676, *p* < 0.001, and *p* < 0.001, respectively).

### Association between serum sodium level and TBM

In the total population, compared with patients in the highest initial sodium quartile (≥ 141 mmol/L), patients in the lowest quartile (< 137 mmol/L) had significantly higher odds of TBM diagnosis, with an OR of 2.0 (95% CI 1.4–2.8, *p* < 0.001) (Table 3). This association was maintained in the propensity-matched population with an OR of 1.8 (95% CI 1.1–2.9, *p* = 0.016).

**Table 3:** Independent association of serum sodium level with TBM compared with VM.

Initial sodium level (mmol/L)			Lowest sodium level (mmol/L)		
	OR (95% CI)	*p* value		OR (95% CI)	*p *value
**Total population of VM and TBM (n = 4896)**					
141≦Na (Q1)	Reference		140≦Na (Q1)	Reference	
139≦Na < 141 (Q2)	0.6 (0.4–1.0)	0.032	138≦Na < 140 (Q2)	1.6 (0.9–3.0)	0.105
137≦Na < 139 (Q3)	0.8 (0.6–1.3)	0.419	135≦Na < 138 (Q3)	2.6 (1.5–4.5)	0.001**
Na < 137 (Q4)	2.0 (1.4–2.8)	< 0.001**	Na < 135 (Q4)	9.6 (5.7–16.2)	< 0.001**
**Propensity-matched population of VM and TBM (n = 558)**					
141≦Na (Q1)	Reference		140≦Na (Q1)	Reference	
139≦Na < 141 (Q2)	0.6 (0.4–1.1)	0.094	138≦Na < 140 (Q2)	1.4 (0.6–2.9)	0.428
137≦Na < 139 (Q3)	0.9 (0.5–1.6)	0.783	135≦Na < 138 (Q3)	2.0 (1.0–4.0)	0.043*
Na < 137 (Q4)	1.8 (1.1–2.9)	0.016*	Na < 135 (Q4)	4.6 (2.4–8.8)	< 0.001**

BM, bacterial meningitis; OR, odds ratio; TBM, tuberculous meningitis; VM, viral meningitis.

Serum sodium levels in the first quartile (Q1), second quartile (Q2), third quartile (Q3), and fourth quartile (Q4).

Analyses were performed with multiple logistic regression tests, controlling for age, sex, cerebrospinal fluid (CSF) white blood cell count, CSF glucose, and CSF protein. Propensity was matched for age, sex, cerebrospinal fluid (CSF) white blood cell count, CSF glucose, and CSF protein.

*p < 0.05, **p < 0.01.

Regarding lowest sodium level, compared with patients in the highest quartile (≥ 140 mmol/L), patients in the lower three quartiles had significantly higher odds of TBM diagnosis both in the total population and the propensity-matched population. In the lowest quartile (< 135 mmol/L), the OR for TBM was 9.6 (95% CI 5.7–16.2, *p* < 0.001) in the total population and 4.6 (95% CI 2.4–8.8, *p* < 0.001) in the propensity-matched population.

The area under the curve for the receiver operating characteristic was 0.795 (*p* < 0.001) with a sensitivity of 75.9% and a specificity of 72.2% for TBM when the lowest sodium level was less than 135.9 mmol/L.

### Association between serum sodium level and mortality at 90 days

In the total population, the mortality rate at 90 days was 9.2% in the TBM group, 14.6% in the BM group, and 1.3% in the VM group (*p* < 0.001). However, there was no association between serum sodium level and mortality at 90 days in the TBM and BM groups (Supplementary Table 1).

## Discussion

In this study, we evaluated the association between hyponatremia and subtype of infectious meningitis: TBM, BM, and VM. The initial serum sodium level was significantly lower in the TBM group than in the BM and VM groups, and it decreased significantly more steeply to lower levels in both the TBM and BM groups compared with the VM group. The lowest serum sodium level was in the order of TBM < BM < VM, and the differences were statistically significant between all subgroups. The participants with lower serum sodium level were more likely to have a diagnosis of TBM rather than VM, and this association was more pronounced for the lowest sodium level than the initial sodium level.

The association between hyponatremia and TBM has been elucidated and is well-known^8,14^. In one study including 76 cases of TBM, hyponatremia was observed in approximately 45% of the cases^12^. The syndrome of inappropriate antidiuretic hormone secretion and cerebral salt wasting are the most likely causes of hyponatremia in patients with TBM and could interact with each other^12^. A recent prospective study conducted in India suggested that cerebral salt wasting is the most common cause of TBM and found a relationship between hyponatremia and presence of basal exudates, implicating pituitary involvement in hyponatremia^12^. Based on these findings, the frequent occurrence of hyponatremia in TBM compared with other meningitis in our study could be attributable to pituitary involvement by basal exudate, which is one of the main prominent features of TBM.

Hyponatremia can develop anytime during TBM^11^ and it worsens cerebral edema, headache, confusion, seizures, and coma^15^ and predicts increased mortality in patients, particularly those with human immunodeficiency virus (HIV) infection and TBM^16^. However, to the best of our knowledge, a cross-sectional or longitudinal comparison according to infectious meningitis etiology has not been performed.

Diagnosing TBM is one of the most challenging differential diagnoses, and the cost of a false-negative diagnosis can be fatal. Subacute clinical onset of TBM, typically with a prodromal period of 2–4 weeks with non-specific symptoms such as fatigue, malaise, myalgia, and fever, often complicates diagnosis of TBM to differentiate it from VM in the early stage^8,17^. Only about 10% of patients with TBM have a history of tuberculosis disease^18^. Furthermore, the CSF profile of TBM typically shows lymphocytic pleocytosis with an average cell count around 200 cells/μL and elevated protein content^8^, which is similar to the profile of aseptic meningitis including VM. Microbiological tests for TBM are insensitive and laborious, and the positive rate of culture in clinical practice is believed to be much lower than measured for various reasons^19^. Our study showed very low positive tuberculosis culture and PCR rates in the CSF of the TBM group, presumably reflecting paucibacillary CSF samples or problems obtaining and processing CSF samples, such as a low volume of CSF, insufficient centrifugation, or examination by inexperienced microscopist in clinical practice^20–22^.

In comparison with BM, TBM shows a slower evolution of meningismus symptoms including headache, fever, vomiting, photophobia, and stiffness of the neck, which usually take more than one week to manifest^23^. In contrast, BM typically shows initial devastating and rapid neurological deterioration with altered consciousness. The CSF profile of TBM also substantially differs from that of BM in that BM typically shows a polymorphonuclear pleocytosis with a cell count greater than 1000 cells/μL, while TBM shows lymphocytic pleocytosis.

Because of the suboptimal sensitivity and specificity of diagnostic tests, treatment often can be started based on a presumptive diagnosis of TBM in the setting of relevant clinical and epidemiologic factors and typical CSF findings. Delays in treatment of TBM, which often are attributable to the discussed clinical and laboratory diagnostic pitfalls and uncertainty, have been associated with high mortality and morbidity rates, including vision loss, cranial nerve dysfunction, hydrocephalus, or vascular complications including stroke and aneurysmal formation and rupture^24–26^.

Serum sodium level evaluation is relatively fast, widely used, and low cost; in addition, it is easy to perform and follow-up and does not require a special facility. Given that TBM is the major disease in developing countries, these advantages of serum sodium evaluation could allow for helpful information in differential diagnosis of meningitis. In this study, we clearly demonstrated the significant association between hyponatremia and TBM compared with other infectious meningitis subtypes. Nevertheless, because hyponatremia is a secondary epiphenomenon of TBM and cannot confirm the presence of *M. tuberculosis*, serum sodium level evaluation cannot be considered a diagnostic method for TBM. Rather, we propose that hyponatremia could provide supportive information for diagnosis of TBM as a surrogate marker.

Our study results require cautious interpretation based on the following limitations. Because of the retrospective study design with data extraction from an integrated big data platform, there were some limitations to the data acquisition. For instance, data on herpes simplex virus, enterovirus, and adenosine deaminase were not available. In contrast, because the data were based on the OCS and its subsequent results, we obtained accurate laboratory results such as serial serum sodium level.

We showed a significant association between hyponatremia and TBM compared with BM and VM, both cross-sectionally and longitudinally. Our results provide useful information in differential diagnosis of TBM from BM or VM so that prompt optimal treatment can be applied.

## Patients and methods

### Participants

We used data from the Clinical Data Warehouse (CDW) database, which is a large, integrated, harmonized database of five tertiary referral medical centers belonging to The Catholic University of Korea, College of Medicine, Seoul, Korea^27^. The information in this database has been collected from electronic medical records (EMR) and order communication system (OCS) since April 1997, the platforms of which are commonly shared by the five hospitals. Briefly, the relational CDW database is composed of a demographics database; a diagnosis database including participant age and date at diagnosis; an admission database including admission dates, discharge dates, and information on inpatients and outpatients; a surgery database; a prescription database including prescribed medications and ordered laboratory tests; a laboratory database with results of ordered tests such as serum sodium level and date of the test; and a histology database including cultures and polymerase chain reaction (PCR) results. Data extraction is performed after anonymization, and researchers are not able to identify and access participant personal information.

The study cohort consisted of patients with TBM, BM, or VM and who were 18 years of age and consecutively admitted to the five hospitals from December 2009 to December 2019. The participants in the TBM group were enrolled when they were diagnosed as possible, probable or definite TBM according to the Marais’ uniform TBM case definition; all patients took anti-tuberculosis medications^28^. We defined a case of BM as patient with headache, mental status changes, and either a positive CSF or blood culture and who was treated with antibiotics. The VM group included patients with no evidence of other meningitis by *M. tuberculosis*, bacterial infection, fungal infection, autoimmune disease, injury, cancer, or certain drugs and who achieved complete recovery with only conservative treatments or had positive CSF results for viral PCR test.

### Study variables

For this study, we obtained demographic information of age at admission for meningitis, sex of the participants, admission date, discharge date, medical department for admission, final diagnosis (by the International Classification of Diseases code), prescribed medications and treatments, results of various laboratory tests available in the database (blood glucose, protein, sodium, procalcitonin, lactate dehydrogenase (LDH) levels, and white blood cell (WBC) count; CSF WBC, glucose, protein, and LDH levels; varicella-zoster virus (VZV) PCR; and tuberculosis Acid-Fast Bacillus (AFB) stain, culture, and real-time PCR in the CSF). In our institution, VZV PCR was performed with the VZV PCR kit provided by BioCore, MagNA Pure 96 (DNA extraction) and a thermal cycler (real-time PCR). AFB stain and culture for mycobacteria were performed using Ziehl–Neelsen staining and Middlebrook 7H9 Broth/Löwenstein–Jensen media.

We obtained serial serum sodium levels at various times points from initial admission to hospital discharge as well as the initial and the lowest sodium levels.

### Standard protocol approvals, registrations, and patient consent

All aspects of this retrospective study were approved by the Institutional Review Board of The Catholic University of Korea (XC19WIDI0113). The requirement for informed consent was formally waived by the Institutional Review Board of The Catholic University of Korea.

### Statistical analyses

All statistical analyses were performed using SPSS for Windows version 17.0 (SPSS Inc., Chicago, IL, USA). Independent t-tests and analysis of variance (ANOVA) were used to compare continuous variables. Pearson’s chi-squared tests and Fisher’s exact test were used to compare categorical variables. The values are expressed as mean ± standard deviation. In addition to analysis of absolute population numbers, we used propensity score matching for the unbalanced numbers in each group and to eliminate the effect of confounding variables. By balancing according to age, sex, and CSF profiles including WBC, glucose, and protein, two similar groups of 276 TBM or VM patients were extracted from the sample. Similarly, comparing TBM versus BM, groups consisting of 105 patients with similar propensity scores were extracted, and 105 propensity-matched patients were extracted when comparing BM versus VM. A cross-sectional comparison of the initial and lowest serum sodium levels was performed using paired t-tests between propensity-matched groups. To assess temporal longitudinal changes in serum sodium level in each meningitis subgroup, repeated-measures ANOVA was performed for the total patients and the propensity-matched population. The initial and lowest serum sodium levels were divided by quartile distribution, and the odds ratios (ORs) were calculated for diagnosis of TBM compared with VM using logistic multivariable regression analysis in the total population and the propensity-matched population. Statistical significance was assumed at a false detection rate less than 5% (*p* < 0.05). The area under the curve for the receiver operating characteristic (ROC) was performed for the sensitivity and specificity of diagnosis of TBM.

### Ethics approval

All procedures performed in studies involving human participants were in accordance with the ethical standards of the institutional committee and the 1964 Declaration of Helsinki and its later amendments or comparable ethical standards.

## Supplementary Information

Supplementary Table 1.

## Data Availability

The de-identified data supporting the findings of this study are available upon reasonable request to the corresponding author.
