# Supplementary material for: The association between serum sodium level and tuberculous meningitis compared with viral and bacterial meningitis
Source: Sci Rep. 2021 May 25;11:10906. doi: 10.1038/s41598-021-90358-5 (PMC8149664; doi:10.1038/s41598-021-90358-5)
Supplement: Supplementary file 1 — Supplementary Table 1. [file 41598_2021_90358_MOESM1_ESM.docx]

| Supplementary table 1 Independent association of serum sodium levels with morality at 90 days in TBM and BM | | | | | |
| --- | --- | --- | --- | --- | --- |
| Initial sodium level (mmol/L) | | | Lowest sodium level (mmol/L) | | |
| TBM (n=295) | | | | | |
|  | OR (95% CI) | *p*-value |  | OR (95% CI) | *p*-value |
| 141≦Na (Q1) | Reference |  | 140≦Na (Q1) | Reference |  |
| 139≦Na<141 (Q2) | 0.5 (0.1-2.1) | 0.322 | 138≦Na<140 (Q2) | 0.5 (0.0-9.7) | 0.677 |
| 137≦Na<139 (Q3) | 0.5 (0.1-2.4) | 0.430 | 135≦Na<138 (Q3) | 0.4 (0.0-4.5) | 0.427 |
| Na<137 (Q4) | 0.9 (0.3-2.5) | 0.820 | Na<135 (Q4) | 1.0 (0.1-8.8) | 0.976 |
| BM (n=130) | | | | | |
|  | OR (95% CI) | *p*-value |  | OR (95% CI) | *p*-value |
| 141≦Na (Q1) | Reference |  | 140≦Na (Q1) | Reference |  |
| 139≦Na<141 (Q2) | 1.1 (0.1-7.7) | 0.951 | 138≦Na<140 (Q2) | 0.5 (0.0-9.3) | 0.651 |
| 137≦Na<139 (Q3) | 0.3 (0.0-3.2) | 0.328 | 135≦Na<138 (Q3) | 0.1(0.0-2.0) | 0.145 |
| Na<137 (Q4) | 0.6 (0.1-2.7) | 0.496 | Na<135 (Q4) | 0.5 (0.1-3.5) | 0.472 |
| Abbreviations: BM = bacterial meningitis; OR = odds ratio; TBM = tuberculous meningitis; VM = viral meningitis.  Serum sodium levels in the first quartile (Q1), second quartile (Q2), third quartile (Q3), and fourth quartile (Q4).  Analyses were performed with multiple logistic regression tests, controlling for age, sex, cerebrospinal fluid (CSF) white blood cell count, CSF glucose and CSF protein.  **P* < 0.05, ***P* < 0.01 | | | | | |
